# Expression of recombinant glutamic acid decarboxylase in insect larvae and its application in an immunoassay for the diagnosis of autoimmune diabetes mellitus

**DOI:** 10.1038/s41598-018-35744-2

**Published:** 2019-01-29

**Authors:** Aldana Trabucchi, Silvina S. Bombicino, Alexandra M. Targovnik, Juan I. Marfía, Adriana V. Sabljic, Natalia I. Faccinetti, Luciano L. Guerra, Ruben F. Iacono, María V. Miranda, Silvina N. Valdez

**Affiliations:** 1 Universidad de Buenos Aires (UBA), Facultad de Farmacia y Bioquímica, Departamento de Microbiología, Inmunología, Biotecnología y Genética, Cátedra de Inmunología, Buenos Aires, Argentina; 2 Consejo Nacional de Investigaciones Científicas y Técnicas (CONICET), Universidad de Buenos Aires, Instituto de Estudios de Inmunidad Humoral Prof. Ricardo A. Margni (IDEHU), Buenos Aires, Argentina; 3 Universidad de Buenos Aires (UBA), Facultad de Farmacia y Bioquímica, Departamento de Microbiología, Inmunología, Biotecnología y Genética, Cátedra de Biotecnología, Buenos Aires, Argentina; 4 Consejo Nacional de Investigaciones Científicas y Técnicas (CONICET), Universidad de Buenos Aires, Instituto de Nanobiotecnología (NANOBIOTEC), Buenos Aires, Argentina

## Abstract

Autoimmune Diabetes Mellitus (DM) is a chronic disease caused by the selective destruction of insulin producing beta cells in human pancreas. DM is characterized by the presence of autoantibodies that bind a variety of islet-cell antigens. The 65 kDa isoform of glutamate decarboxylase (GAD65) is a major autoantigen recognized by these autoantibodies. Autoantibodies to GAD65 (GADA) are considered predictive markers of the disease when tested in combination with other specific autoantibodies. In order to produce reliable immunochemical tests for large scale screening of autoimmune DM, large amounts of properly folded GAD65 are needed. Herein, we report the production of human GAD65 using the baculovirus expression system in two species of larvae, *Rachiplusia nu* and *Spodoptera frugiperda*. GAD65 was identified at the expected molecular weight, properly expressed with high yield and purity in both larvae species and presenting appropriate enzymatic activity. The immunochemical ability of recombinant GAD65 obtained from both larvae to compete with [^35^S]GAD65 was assessed qualitatively by incubating GADA-positive patients’ sera in the presence of 1 μM of the recombinant enzyme. All sera tested became virtually negative after incubation with antigen excess. Besides, radiometric quantitative competition assays with GADA-positive patients’ sera were performed by adding recombinant GAD65 (0.62 nM–1.4 µM). All dose response curves showed immunochemical identity between proteins. In addition, a bridge-ELISA for the detection of GADA was developed using *S*. *frugiperda-*GAD65. This assay proved to have 77.3% sensitivity and 98.2% of specificity. GAD65 could be expressed in insect larvae, being *S*. *frugiperda* the best choice due to its high yield and purity. The development of a cost effective immunoassay for the detection of GADA was also afforded.

## Introduction

Type 1 Diabetes Mellitus (T1DM) is a widespread disease that may lead to the development of severe clinical conditions, such as ketoacidosis, retinopathy, neuropathy, nephropathy and death due to severe metabolic imbalance. The global incidence of T1DM is increasing by approximately 3% per year, with patients requiring life-long insulin replacement therapy^1^. T1DM is a chronic disease caused by the selective destruction of insulin producing beta cells of the pancreas, mediated by a clinically silent autoimmune process^2,3^. Both humoral and cellular immune responses are associated with T1DM, with autoantibodies that bind a variety of islet-cell antigens. Current diabetes studies are focused on the prediction and prevention of insulin deficiency in T1DM. To that end, large-scale screening for autoantibodies must be carried out.

A major autoantigen recognized by these autoantibodies is an islet-cell protein identified as the 65 kDa isoform of glutamic acid decarboxylase (GAD65). This enzyme catalyzes the decarboxylation of glutamic acid to γ-aminobutyric acid (GABA) and CO_2_^4–8^. Autoantibodies to GAD65 (GADA) are a valuable humoral marker that can be used both to classify and monitor the progression of the disease^9^. The other autoantibodies present in autoimmune DM are: insulin/proinsulin autoantibodies (IAA/PAA), insulinoma-associated tyrosine phosphatase 2 autoantibodies (IA-2A) and zinc transporter isoform 8 autoantibodies (ZnT8A). When assay thresholds for IAA/PAA, GADA, IA-2A and ZnT8A are set at the 99th percentile of controls, approximately 98% of children with new-onset diabetes are found to express at least one of these autoantibodies^10^. In addition, GADA are considered predictive markers when tested in combination with other disease-specific autoantibodies^11^, such as those of autoimmune tyroid disease, celiac disease, Addison’s disease and vitiligo^12^.

Therefore, in order to produce reliable immunochemical tests for large scale screening of population deemed at risk due to a family history of autoimmune diabetes, and/or other genetic factors, large amounts of properly folded human GAD65 are needed. In addition, it is interesting to explore its potential as tolerogen in the prevention of T1DM^13–15^.

Isolating GAD65 in high amounts from animal tissues is almost impracticable; therefore, the enzyme should be obtained as a recombinant protein. Native GAD65 can be produced in baculovirus-infected *Sf*9 cells^16,17^, however, its expression in baculovirus-infected larvae has not been reported.

The baculovirus-insect expression system is highly chosen when there is a need to express eukaryotic proteins taking into account their native environment. This almost occurs when the desired protein is destined to biological or pharmaceutical purposes^18^. However, the production of recombinant proteins in insect cell culture has a big disadvantage: industrial scale production has high costs^19^. As an alternative, the use of insect larvae as bio-factories for the production of recombinant proteins is a more economic strategy, mainly because the need of tissue-culture specialized facilities is minimal^20,21^. The order Lepidoptera (butterflies and moths) is a large group of insects comprising more than 100,000 species. *Spodoptera frugiperda* (*S*. *frugiperda*) and *Rachiplusia nu* (*R*. *nu*) are two of the most abundant and widely distributed lepidopteran species in Argentina.

In this report, we describe the production of human GAD65 using a baculovirus expression system in two species of larvae, *R*. *nu* and *S*. *frugiperda*. Our aim was to obtain large quantities of properly folded and immunochemically active human GAD65 to be used in the development of immunochemical tests for the screening of autoimmune diabetes.

## Material and Methods

### Materials and reagents

Sf900II insect tissue culture media, the *S*. *frugiperda Sf*9 cell line, Cellfectin® and penicillin/streptomycin (ATB/ATM) were from Invitrogen Life Technologies (Gaithersburg, MD, USA). Larvae were obtained from AgIdea S. A. (Pergamino, Buenos Aires, Argentina). Fetal bovine serum (FBS) was from Internegocios (Buenos Aires, Argentina). *Autographa californica* nuclear polyhedrosis virus (AcMNPV), pAcGP67-B vector, Agarplaque Plus and BaculoGold Bright were from BD Biosciences Pharmingen (San Diego, CA, USA). Disposable materials were from Nunc International (Naperville, IL, USA). The Low Molecular Weight Calibration kit (14.4–97.0 kDa), used in SDS-PAGEs and western blots, was from GE Healthcare Life Science (Chicago, IL, USA). Antibodies against His_6_ were from BD Biosciences Pharmingen (San Diego, CA, USA). Monoclonal antibodies to GAD65 (GAD6) were obtained from the supernatant of a hybridoma cell culture purchased from the Developmental Studies Hybridoma Bank. Peroxidase-conjugated goat antibodies to mouse IgG were from Jackson ImmunoResearch Laboratories, Inc. (West Grove, PA, USA). Other reagents were of analytical grade.

### Human sera collection

Blood samples were collected from patients after overnight fasting and the corresponding sera were stored at −20 °C until assayed. Sera were obtained from selected diabetic patients with a wide range of GADA titers. Sera were selected among the samples collected in our laboratory during the routine detection of autoantibodies (Servicios Tecnológicos de Alto Nivel, STAN-CONICET).

Control sera were obtained from 56 healthy subjects without personal or family history of autoimmune disease. This work was performed with the approval of the Ethical Committee of José de San Martín Clinical Hospital, Buenos Aires, Argentina.

All experiments were done in accordance with the relevant guidelines and regulations.

Written informed consent was obtained from all participants.

### Virus production

The cDNA encoding the full-length human GAD65 bearing a histidine-hexapeptide (His_6_) tail at the N-terminus (synthesized by GenScript Corporation, Piscataway, NJ, USA; www.GenScript.com) was directly cloned into the pAcGP67-B transfer vector downstream the baculovirus polyhedrin promoter and the gp67 viral signal peptide sequence, which targets the recombinant protein for secretion (pAcHis_6_-GAD65). One million *Sf*9 cells were co-transfected with 0.25 μg pAcHis_6_-GAD65 and 1 μg linearized BaculoGold Bright DNA in the presence of Cellfectin® according to standard baculovirus protocols^22,23^. BaculoGold Bright DNA contains the GFP gene. After a 4-day incubation at 27 °C, the cell culture supernatant was collected and centrifuged at 3,000 × g for 10 min. The co-transfection efficiency was determined by measuring the GFP expression under UV light. The recombinant baculovirus was named AcHis_6_-GAD65. After 4 days of infection, supernatants of co-transfection were used to infect monolayer cultures at a multiplicity of infection (MOI) of 0.02 (first amplification step of the viral stock). This first amplification supernatant was used to perform the second amplification. After three amplification steps, the virus titer was determined by a plaque assay (8.6 × 10^7^ pfu/mL)^23^. This amplified virus stock was used for the production of the recombinant protein in further experiments. Baculovirus was stored at 4 °C until used for the expression of human GAD65 in insect cells and larvae.

### Insect cells and larvae

*Sf*9 cell suspension cultures were grown in sterile Erlenmeyer flasks under continuous shaking at 100 × g in Sf900II medium supplemented with 1% (v/v) fetal bovine serum (FBS) and 1% antibiotic-antimycotic solution at 27 °C. All assays were carried out using *Sf*9 cells from suspension cultures in log-phase growth with 95–99% viability. *R*. *nu* and *S*. *frugiperda* larvae were obtained from a laboratory colony and reared individually in standard 6-well plates on an artificial high-wheat germ diet^24^ at 23–25 °C in a 70% humidified chamber, with a 16:8 h light/dark photoperiod.

Controls were done with either non-infected cells and larvae, or with cells and larvae infected with baculovirus expressing only the GFP.

### Recombinant GAD65 expression and purification from cell cultures

The expression of GAD65 was first evaluated at different MOI (0.5, 1, 2, 4 and 8 pfu per cell) on 6 × 10^5^ *Sf*9 cells/mL. After infection, cells were incubated in the dark at 27 °C. To study the expression among the different days post-infection (dpi), 1 mL samples were collected each day until 7 dpi. Culture supernatants were separated from the cells by centrifugation at 10,000 × g for 10 min. Sedimented cells and supernatants were then analyzed by SDS-PAGE and WB with monoclonal antibodies against GAD65 (GAD6). The best expression conditions were selected.

The expression of GAD65 was performed at a MOI = 0.5 on 2 × 10^6^ *Sf*9 cells/mL. Infections were incubated in the dark at 27 °C for 4 days and cells were collected by centrifugation at 10,000 × g for 10 min. Sedimented cells were resuspended in 1 mL of lysis buffer (20 mMTris/HCl, pH 7.4) and sonicated over crushed ice in the presence of 0.05 M EDTA, protease inhibitors (0.1% w/v aprotinin and 2 mM phenylmethylsulfonylfluoride –PMSF-), 100 mM 2-aminoethyl isothiouronium bromide –AET- an antioxidant agent, and 0.2 mM pyridoxal 5-phosphate –PLP- GAD65 cofactor. After sonication, Triton-X 100 was added to a final concentration of 1% v/v and incubated for 15 min at 0 °C under constant shaking. The soluble intracellular fraction was then separated by centrifugation at 20,000 × g for 30 min. The supernatant was adjusted to 20 mM NaH_2_PO_4_, pH 8, 0.5 M NaCl and then directly applied onto a chelating Sepharose-FF column charged with nickel ions. After 1 h incubation at 4 °C, the suspension was poured into a 1.5 cm × 5.0 cm column and washed twice with 2 mL of 20 mM NaH_2_PO_4_, pH 8, 0.5 M NaCl. The recombinant GAD65 protein was eluted with 500 mM imidazole and then stored with a mixture of 50% v/v glycerol, 0.2 mM PLP, 0.05% v/v Tween 20 and 0.1% w/v aprotinin.

### Recombinant GAD65 expression and purification from larvae

Larvae of *R*. *nu* and *S*. *frugiperda* were injected into the hemocele with 50 μL recombinant baculovirus (10^7^ pfu/mL). Control larvae were either uninfected or infected with another baculovirus expressing GFP. Once infected, they were incubated at 24 °C.

For all experiments, fifth-instar larvae were sedated by incubation on ice for 5 min before injection.

Four days after infection, larvae were harvested and their weights determined before individual homogenization in the presence of 1 mL of lysis buffer per larva (10 mM Tris-Cl, pH 8.0, 1 mM EDTA, 0.5 mM EGTA, 1% NP-40, 0.1% sodium deoxycholate, 0.1% SDS, 140 mM NaCl) using a mortar and pestle, and sonicated in the presence of protease inhibitors (0.1% v/v aprotinin and 2 mM PMSF), 100 mM AET, 0.2 mM PLP and 1 mM DTT. After sonication, Triton X-100 was added to a final concentration of 5%, and the mixture was incubated for 30 min at 4 °C under constant shaking. The soluble fraction was separated by centrifugation at 12,000 × g for 30 min at 4 °C.

The larvae soluble fraction was adjusted to 20 mM NaH_2_PO_4_, pH 8, 0.5 M NaCl and 50 mM imidazole by exclusion chromatography and then directly applied into a chelating Sepharose-FF column charged with nickel ions. After 1 h incubation at 4 °C, the suspension was poured into a 1.5 cm × 5.0 cm column and washed twice with 2 mL of 20 mM NaH_2_PO_4_, pH 8, 0.5 M NaCl and 50 mM imidazole. Bound proteins were eluted with 5 vol elution buffer (20 mM NaH_2_PO_4_, pH 8, 0.5 M NaCl, 500 mM imidazole). Purified proteins were stored at −20 °C with a mixture of 50% v/v glycerol, 0.2 mM PLP, 0.05% v/v Tween 20 and 0.1% w/v aprotinin.

### Sodium dodecyl sulphate-polyacrylamide gel electrophoresis and western blot analysis

Protein fractions were separated by 10% SDS-PAGE under reducing conditions, followed by Coomassie Brilliant Blue R-250 staining^25^. For detection by WB^26^, protein bands were transferred onto nitrocellulose membranes and unoccupied binding sites were blocked by incubating with 3% w/v skim milk in Tris buffer saline (TBS: 0.05 M Tris-HCl, 0.15 M NaCl, pH 7.5) for 2 h at room temperature (RT). After 3 washing steps with TBS, membranes were incubated ON at 4 °C with either a monoclonal antibody to GAD65 diluted 1/200 or a monoclonal antibody to His_6_, diluted1/3000 both in 3% w/v skim milk, 0.05% v/v Tween 20 in TBS (TBS-MT), and then washed five times with 0.05% v/v Tween 20 in TBS (TBS-T). Bound antibodies were visualized by incubation with peroxidase-conjugated goat antibodies to mouse IgG diluted 1/2000 in TBS-MT, followed by the addition of α-chloronaphthol (Sigma-Aldrich, Inc., St Louis, MO) and 10 vol. H_2_O_2_.

### GAD65 enzymatic activity measurement

The detection of the enzymatic activity of GAD65 of selected samples was performed by a colorimetric assay in a 96-well microplate^27^. This method is based on the detection of the pH increase due to the consumption of protons occuring as the GAD65-catalyzed reaction proceeds. GAD65 is a PLP-dependent enzyme that catalyzes the α-decarboxylation of L-glutamatic acid to γ-aminobutyric acid (GABA). The decarboxylation of glutamic acid irreversibly incorporates a proton into GABA during CO_2_ release.

The reaction medium in each well consisted of 200 μL of 50 mM acetate buffer, pH 4.8 containing 70 μM bromocresol green, 1 mM PLP and 4 μL of glutamate (1 M in 50 mM acetate buffer). Ten μL of each sample (between 0.3 to 0.6 mg/ml of protein) were added to different wells. The plate was quickly placed in a microplate reader (Varioskan Lux, Thermo Scientific, Waltham, MA, USA) and shaken for 10 seconds to ensure complete mixing. The increase in absorbance at 620 nm was monitored at 40 °C. Optical density values were recorded every 30 s for 10 min. Activities were calculated from the slopes measured in the linear portion of the curve, typically over the first 300 s. The total amount of protein in each well was taken into account to calculate specific activity (μmol min^−1^ mg^−1^). Calculations were done as previously reported^27^.

Eluates of larvae infected with other baculovirus served as negative controls.

### Immunochemical characterization of recombinant GAD65

#### Production of a [^35^S]GAD65 tracer

As previously described in our earlier work^28^, with some modifications, the tracer [^35^S]GAD65 was obtained by *in vitro* transcription/translation of cDNA encoding the human GAD65 using a rabbit reticulocyte lysate system (Promega, Madison, WI, USA) in the presence of [^35^S]-methionine (New England, Nuclear, Boston, MA, USA), according to the manufacturer’s instructions. Translation products were diluted in RBA buffer (0.02 M Tris–HCl, 0.15 M NaCl, pH 7.4, 0.1% v/v Tween 20) and applied to a PD10 column (Pharmacia-LKB Biotechnology, Uppsala, Sweden) in order to remove free [^35^S]-methionine.

#### Inhibition assay

As previously described in our earlier work^29^ for another recombinant autoantigen related to DM, the ability of GAD65 to compete with human [^35^S]GAD65 was assessed qualitatively by incubating 2.5 µL sera from 8 type 1 diabetic patients with 10,000 cpm of tracer in the presence of 1 μM purified protein, either from *R*. *nu*, *S*. *frugiperda* or *Sf*9 cells, in a final volume of 60 µL. Twenty six control sera were also analyzed in order to establish a cut off value. After ON incubation, 50 μL of 40% v/v proteinA-Sepharose 4B FF (GE Healthcare Biosciences, Uppsala, Sweden) in RIA buffer (0.02 M Tris-HCl, 0.15 M NaCl, 0.1% Tween 20, pH 7.4) were added and incubated for 2 h at RT on an end-over-end shaker. Subsequently, samples were allowed to settle and the supernatants were discarded in order to isolate immunocomplexes. Pellets were washed three times with 200 µL of RIA buffer and once with 200 µL of 0.2 M NaCl in RIA buffer. Finally, pellets were suspended in 100 µL of 1% SDS and supernatants were carefully transferred to vials for scintillation counting (1 min/tube). Results for each sample were calculated as B%.

#### Radioimmunoassay protocol

Quantitative competition assays were performed by a standard radioimmunoassay (RIA), as previously described in our earlier work^28^. The RIA was carried out by incubating 2.5 µL of 5 GADA-positive type 1 diabetic patient sera with 10,000 cpm of [^35^S]GAD65 in the presence of serial concentrations (0.62 nM-1.4 µM) of purified *R*. *nu*-GAD65 and *S*. *frugiperda-*GAD65 in a final volume of 60 µL. The following steps of the assay are the same as those described previously for the qualitative assay. Results for each sample were calculated as B%. Inhibitory dose-response curves [log (inhibitor) *vs*. response - Variable slope (four parameters)] were fitted to the mathematical function:

$${\rm{B}}/{{\rm{B}}}_{0}={\rm{B}}/{{\rm{B}}}_{0{\rm{\min }}}+({\rm{B}}/{{\rm{B}}}_{0{\rm{\max }}}-{\rm{B}}/{{\rm{B}}}_{0{\rm{\min }}})/(1+{10}^{[(\mathrm{log}{\rm{IC50}}-\mathrm{log}{{\rm{His}}}_{6}{\rm{GAD}}65{\rm{dose}})\ast {\rm{Hill}}{\rm{Slope}}]})$$

where B corresponds to B% measurements, B_0_ is the B% at zero concentration of unlabelled antigen, B/B_0min_ and B/B_0max_ are the minimal and maximal response, respectively and the parameter IC_50_ represents the concentration of GAD65 that gave a response half between B/B_0min_ and B/B_0max_. The Hill slope describes the steepness of the family of curves.

### *S*. *frugiperda*-GAD65 application in immunoassay for the detection of GADA

The coating buffer was PBS, the blocking buffer was 2% w/v skim milk in PBS, the washing buffer was PBS containing 0.05% v/v Tween 20 (PBS-T). Reagent dilutions were prepared in 2% w/v skim milk in PBS-T (PBS-MT). Avidin–Horseradish Peroxidase (HRP) was purchased from Jackson ImmunoResearch Laboratories, Inc. (West Grove, PA, USA). The 3,3′,5,5′-tetramethyl-benzidine/H_2_O_2_ mixture (Single Component TMB Peroxidase EIA Substrate Kit, BioRad, Hercules, CA, USA) was employed as the chromogenic substrate. Except otherwise indicated, incubations were performed at RT, washing steps were performed with PBS-T and 50 μL per well were added in each incubation step.

#### Biotinylation of *S. frugiperda*-GAD65

Two mL of the purified *S*. *frugiperda*-GAD65 were subjected to buffer exchange to PBS using a ZEBA desalt spin column (Pierce Biotechnology, Rockford, IL, USA) according to the manufacturer’s instructions. The desalted protein was then incubated for 2 h at 0 °C with a 20-fold molar excess of sulfo-NHS-biotin (Pierce Biotechnology, Rockford, IL, USA). The free biotin was removed using another ZEBA desalting spin column.

#### Bridge-ELISA protocol (b-ELISA)

The protocol employed was based on that previously described^30^, with minor modifications. Briefly, polystyrene microplates (Maxisorp, NUNC, Rorkilde, Denmark) were coated ON at 4 °C with 0.15 µg of purified *S*. *frugiperda*-GAD65 per well, washed three times with PBS, blocked for 1.5 h with 200 µL of blocking solution per well, and washed five times. Samples were added in duplicate and microplates were incubated for 1 h. Plates were then washed five times and 22.0 ng of *S*. *frugiperda*-GAD65-biotin per well were added. After another1 h incubation period, plates were washed five times and the bound *S*. *frugiperda*-GAD65-biotin was detected by the addition of Avidin-HRP diluted 1/2000. After 1 h incubation, microplates were washed five times plus one final washing step with 200 μL of PBS. The chromogenic substrate was then added and plates were incubated in the dark. The color reaction was stopped with 4N H_2_SO_4_.

### Statistical analysis

The same as described in Guerra *et al*.^28^, the immunochemical identities and parallelism between inhibitory dose-response curves obtained with *Sf*9-GAD65, *R*. *nu*-GAD65 and *S*. *frugiperda*-GAD65 were analyzed by comparing B/B_0min_, B/B_0max_, Hill slope and IC_50_ values using the extra sum-of-squares F test comparison method. The statistical significance when comparing IC_50_ was determined using the Mann-Whitney *U*-test. The normal distribution of data was assessed by the D’Agostino and Pearson omnibus normality test. In order to remove outliers from normally distributed healthy control individuals, the Rout test was performed. The performance of the b-ELISA was analyzed by determining the area under the curve (AUC) of receiver operating characteristic (ROC) curves. The Spearman coefficient (r_s_) was calculated to evaluate the inter-assay correlation. The kappa index was used to measure the [strength of agreement] = concordance between the b-ELISA and RBA. A kappa value of 0.01–0.20 was indicative of slight agreement; 0.21–0.40, fair agreement; 0.41-0.60, moderate agreement; 0.61–0.80, substantial agreement; and 0.81–1.00, almost perfect or perfect agreement^31–34^.

All calculations were performed using GraphPad Prism version 6.01 for Windows (GraphPad Software, San Diego, CA, USA, www.graphpad.com). A p value < 0.05 was considered statistically significant.

## Results

### Expression of Human GAD65 in *Sf*9 Insect Cells

**Figure 1:**
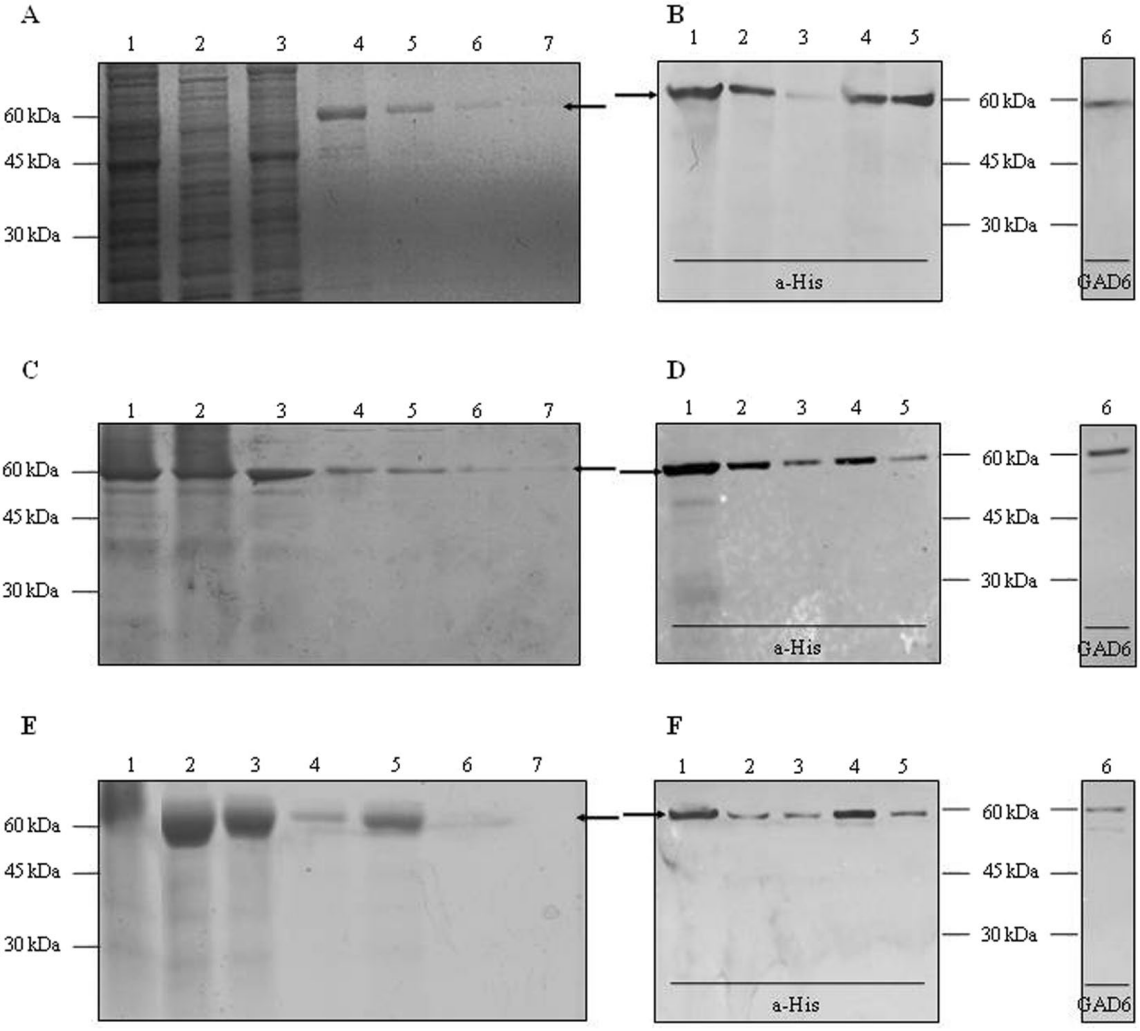
Expression and purification of GAD65. Expression and purification in *Sf*9-cells (**A**,**B**), in *R*. *nu* (**C**,**D**) and in *S*. *frugiperda* (**E**,**F**). (**A**,**C**,**E**) SDS-PAGE (10.0% T 6.0% C, 1.5 mm, under reducing conditions, stained with Coomassie Brilliant Blue R-250); (**B**,**D**,**F**) WB revealed with monoclonal antibodies to either His_6_ or GAD65 as primary antibody. (**A**,**C**,**E**) Samples: 1. Total soluble fraction, 2. Unbound material, 3. Wash step, 4–7. Consecutive eluates of purified GAD65. (**B**,**D**,**F**) Samples: 1. Total soluble fraction, 2. Unbound material, 3. Wash step, 4–6. Consecutive eluates of purified GAD65. Arrows indicate the electrophoretic mobility of GAD65. Full-length WB are presented in Supplementary Fig. 1.

The recombinant human GAD65 was expressed in *Sf*9 cells. Efficient *Sf*9-GAD65 production was achieved after 4 days of infection, yielding 3.60 mg of 95% pure GAD65/L culture medium. SDS-PAGE and WB analyses of total cell lysates and the different purification steps (Fig. 1A,B), with monoclonal specific antibodies to His_6_, showed one band with the expected molecular weight (~66 kDa) for the full-length engineered protein. The identity of the protein was confirmed by WB after incubation with monoclonal antibodies to GAD65 (GAD6).

Despite having the gp67 signal peptide, the expressed protein was not efficiently exported to the supernatant, but remained inside the cells. Consequently, different lysis protocols were tested to achieve a suitable recovery of native GAD65 in the supernatants.

### Expression of Recombinant Human GAD65 in Insect Larvae

Recombinant AcHis_6_-GAD65 baculovirus was also used to infect insect larvae. *R*. *nu* and *S*. *frugiperda* were infected by intrahemocele injection with 50 μL of 10^7^ pfu/mL. After 4 days of infection, both species of larvae were able to express GAD65 with the expected molecular weight of 66 kDa, as demonstrated by SDS-PAGE and WB analyses (Fig. 1C,D for *R*. *nu*-GAD65, and Fig. 1E,F for *S*. *frugiperda*-GAD65). GAD65 was efficiently extracted using a lysis buffer containing a mixture of ionic and non-ionic detergents. The expression levels of GAD65 in *R*. *nu* larvae was 1.06 mg of protein per g of larva, with 95% of purity, according to a densitometric analysis; while in *S*. *frugiperda* larvae, the expression of GAD65 reached 5.70 mg of protein per g of larva with 97% of purity.

### Enzymatic Activity

In order to demonstrate that the structure of recombinant human GAD65 expressed by baculovirus insect larvae was correctly folded, at least in the proximity of the catalytic site, the enzymatic activity was determined by measuring the change in color of bromocresol green, from green to blue. This color change is proportional to the pH increase that occurs as GAD65 catalyzes the α-decarboxylation of L-glutamic acid to GABA by consuming protons of the reaction medium. *R*. *nu*-GAD65 and *S*. *frugiperda*-GAD65 exhibited a specific activity of 190 and 72.8 μmol min^−1^ mg^−1^, respectively. Recombinant GAD fused to thioredoxin (TrxGAD)^35^ expressed in *E*. *coli* was used as a standard protein obtaining an enzymatic activity value of 96 μmol min^−1^ mg^−1^. All these data confirm that the active site of recombinant GAD65 was correctly folded.

### Immunochemical Characterization of Recombinant GAD65

**Figure 2:**
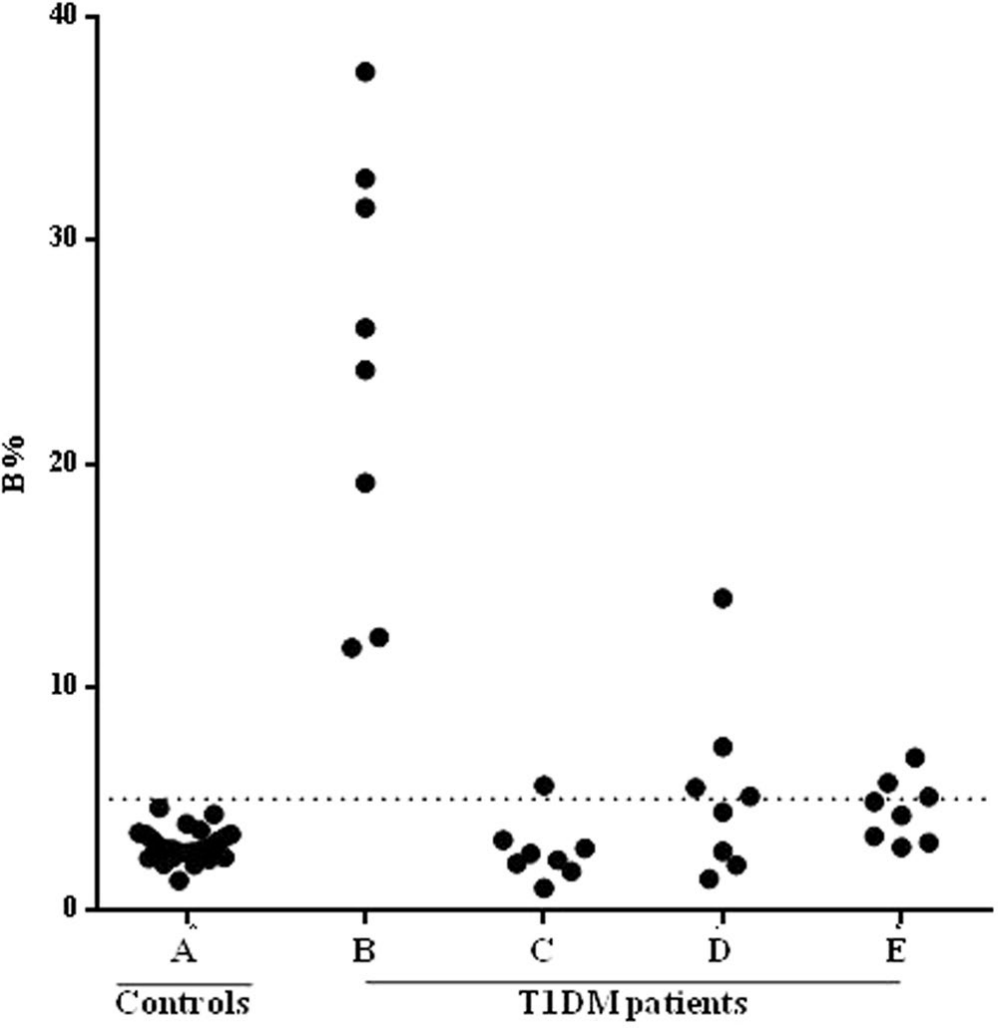
Inhibition capacity of GAD65 in the RBA of 8 GADA positive sera. (**A**) Binding of [^35^S]GAD65 to 26 control human sera, (**B**) binding to GADA-positive sera, (**C**) binding to GADA-positive sera in the presence of 1 μM of *Sf*9-GAD65, (**D**) binding to GADA-positive sera in the presence of 1 μM of *R*. *nu*-GAD65, (**E**) binding to GADA-positive sera in the presence of 1 μM of *S*. *frugiperda*-GAD65. Binding is expressed as bound % (B%), the cut-off value is shown as a dotted line.

To test the ability of *R*. *nu*-GAD65 and *S*. *frugiperda*-GAD65 to react with GADA, radiocompetition assays were performed. Sera from 8 type 1diabetic patients that scored positive by RBA were used. All sera tested were GADA-positive with a B% that ranged from 9.12% to 37.56%. In addition, 26 control sera from healthy subjects were used to determine a cut off value (B% 5.03%) and to rule out interferences generated by the sample matrix. The assay was carried out in parallel in the presence of ~1 μM GAD65 from *R*. *nu*, *S*. *frugiperda* and *Sf*9, for comparison. All 8 positive sera became virtually negative under this condition of cold antigen excess, with a B% range from 1.43% to 13.99%, 2.86% to 6.87% and 1.03% to 5.62%, respectively (Fig. 2). These assays showed that *R*. *nu*-GAD65 and *S*. *frugiperda*-GAD65, as well as *Sf*9-GAD65, inhibit the binding of GADA to $[$ ^35^S]GAD65.

**Figure 3:**
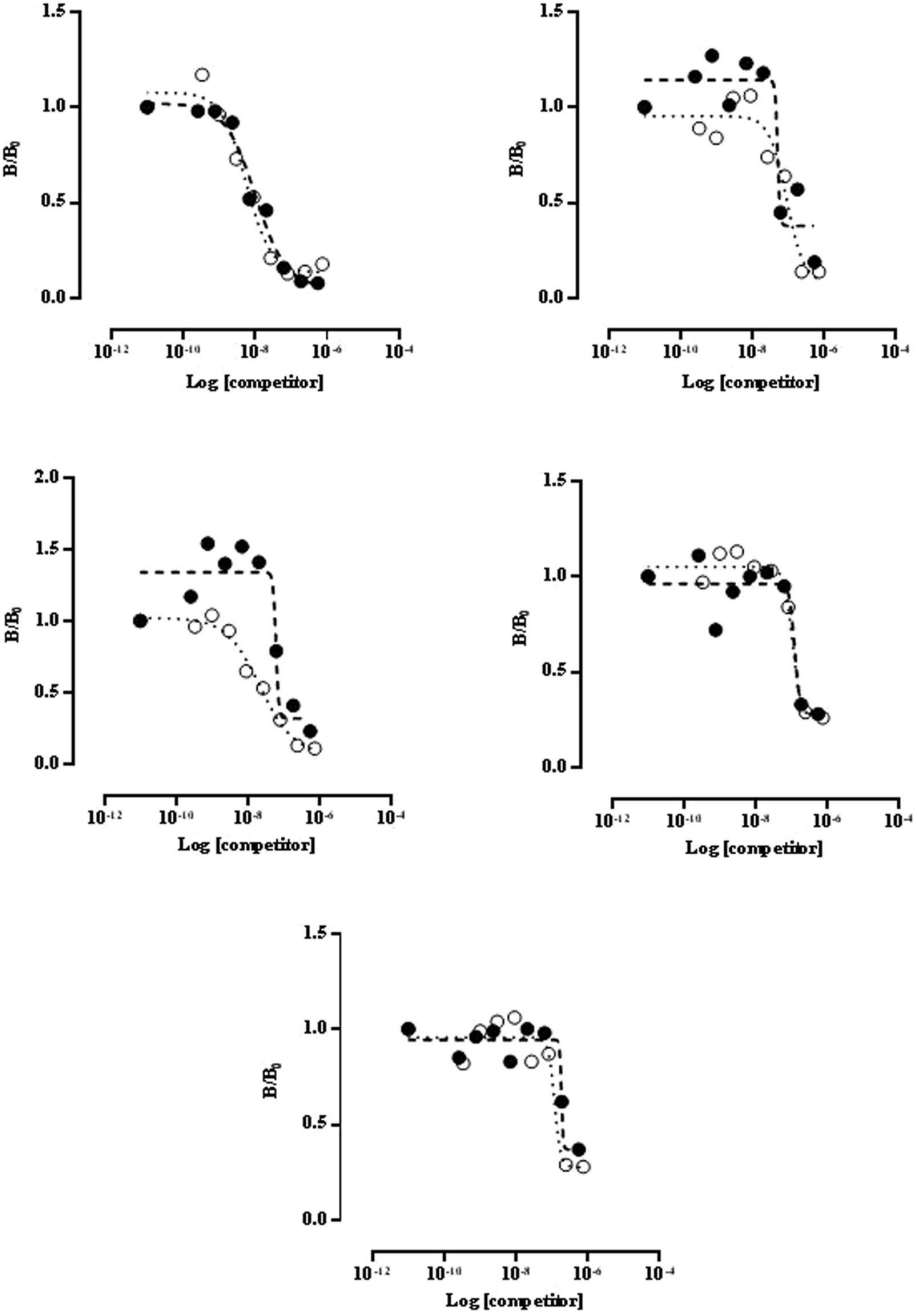
Quantitative competition radioimmunoassays performed with 5 GADA-positive sera. *R*. *nu*-GAD65 (closed circle) and *S*. *frugiperda*-GAD65 (open circle) were used as alternative competitors to displace the [^35^S]GAD65 tracer.

In order to study the immunochemical behavior of GAD65 obtained from larvae, dose-response curves with 5 sera from GADA-positive diabetic patients were performed by RIA, using [^35^S]GAD65 as tracer and variable concentrations of *R*. *nu*-GAD65 and *S*. *frugiperda*-GAD65 (Fig. 3). All dose-response curves (B/B0 *vs*. log [GAD65]) demonstrated comparable GAD65 immunoreactivities with sera. The GAD65 concentration that caused 50% inhibition (IC_50_) was calculated for each serum. This concentration was similar for 4 out of the 5 sera analyzed, ranging from 1.07 × 10^−7^ M to 7.21 × 10^−9^ M. These values are comparable to those reported for pure TrxGAD produced in *E*. *coli*^35^ and to the value estimated for pure GAD65 previously produced in *Sf*9 insect cells^17^. In addition, no significant differences were found between the values obtained from *R*. *nu*-GAD65 and *S*. *frugiperda*-GAD65 (p < 0.05). These data suggest that the recombinant protein expressed in both larvae are immunochemically identical.

### Detection of GADA by ELISA using *S*. *frugiperda*-GAD65

**Figure 4:**
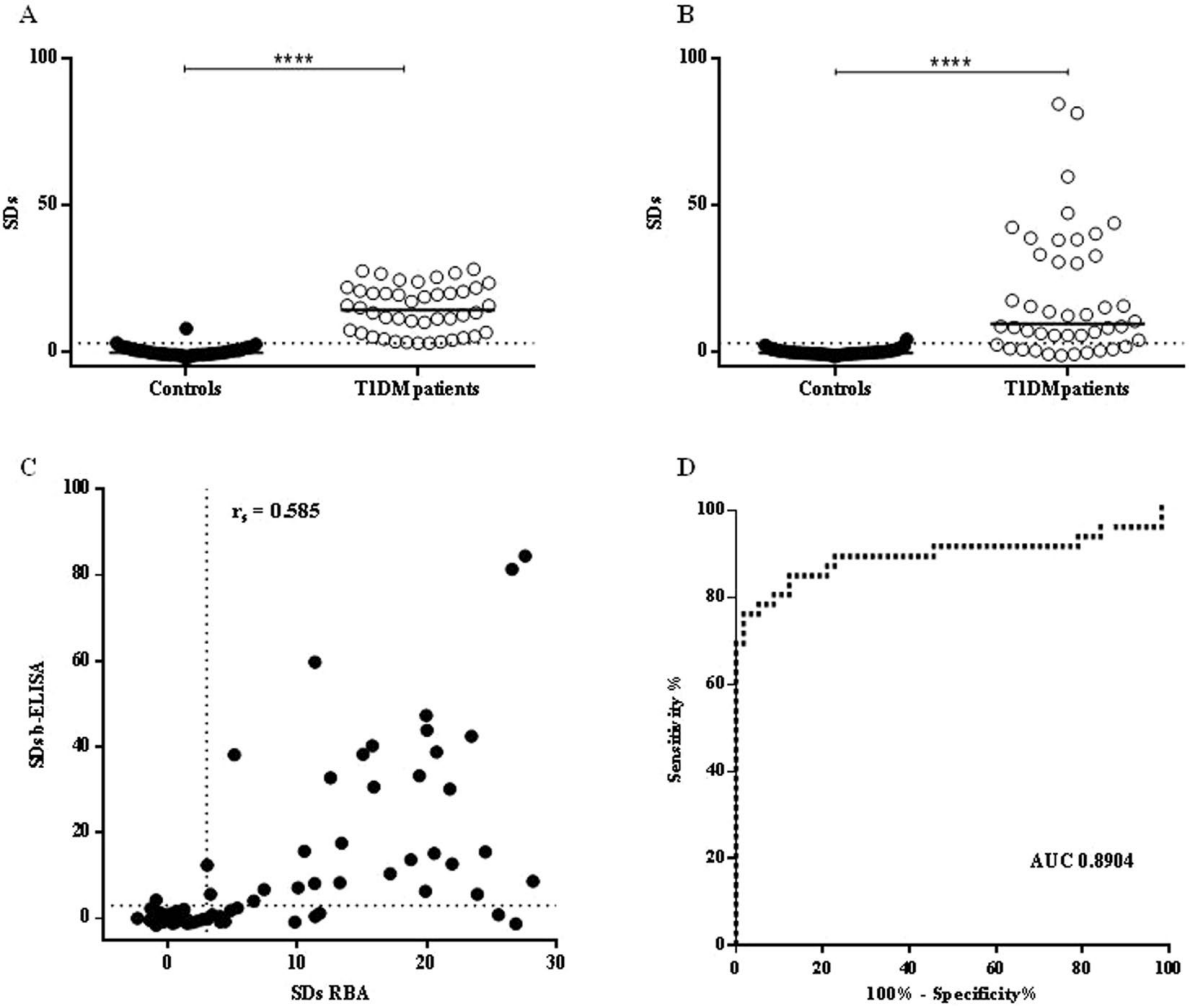
Immunoassays for the assessment of GADA in healthy control and type 1 diabetic patients’ sera. Results, expressed as SDs, were obtained by RBA (**A**) and b-ELISA (**B**). The cut-off value for each assay is indicated as a dotted line and medians for each population are indicated as a full line (****p < 0.0001). (**C**) Correlation analysis for the detection of GADA by RBA and b-ELISA, r_s_ is included. (**D**) ROC curve analysis of b-ELISA, AUC is included.

Forty four patient sera displaying a wide range of GADA titers were tested in parallel by RBA and bridge-ELISA (b-ELISA) for the detection of GADA. The specificity was calculated as 100% minus the percentage of false positives (obtained with healthy control individuals, n = 56). In order to obtain normally distributed values for healthy control individuals, an outlier removal was needed (Rout test, Q = 1%); however, these outliers were included in all plots and in specificity calculations, as they were considered false positives. When analyzed by RBA, a median SDs of 14.25 was obtained for the 44 diabetic patients sera analyzed, ranging from 3.04 to 28.21; cut-off value for positivity SDs = 3.0 (Fig. 4A and Table 1). With this analysis, a specificity of 96.4% was obtained and healthy control individuals differed significantly from diabetic patients (p < 0.0001).

**Table 1:** Analytical parameters of RBA and b-ELISA.

	Median (SDs)	Range (SDs)	Specificity^a^ (%)	Analytical Sensitivity^b^ (%)
RBA	14.25	3.04–28.21	96.4	—
b-ELISA	9.55	−(1.21)–84.47	98.2	77.3

^a^100 minus the percentage of false positives.

^b^Percentage of patients RBA positive that were positive by b-ELISA.

Out of the 44 diabetic GADA-positive patients by RBA, 34 (77.3%) were positive by b-ELISA, with SDs ranging from −1.21 to 84.47 and a median of 9.55; with a cut-off value for positivity SDs = 3.0. These results indicated that b-ELISA had an analytical sensitivity of 77.3% for this sera collection (percentage of patients RBA positive that were positive by b-ELISA) and 98.2% specificity (Fig. 4B and Table 1). In addition, the dynamic range of b-ELISA was significantly wider than that of RBA. Despite the differences in the physical–chemical principles of both techniques, a significant correlation was found between them (Spearman’s coefficient, r_s_ = 0.5850; p < 0.0001). Figure 4C shows a statistical comparison of RBA and b-ELISA from diabetic patients and control healthy individuals; the concordance between the two immunoassays was 96.7%, with a kappa statistic of 0.9302, representing an excellent agreement. Moreover, the performance of b-ELISA was analyzed using ROC analysis, obtaining an AUC of 0.8904 (Fig. 4D).

## Discussion

Glutamic acid decarboxylase is a major autoantigen in autoimmune DM. The presence of specific autoantibodies (GADA) in diabetic patients and the population at risk is the serological evidence of a silent autoimmune process against pancreatic beta cells. This feature turns GADA into a promising humoral immune marker for autoimmune DM. GADA is one of the earliest detectable islet cell autoantibodies, and is considered highly predictive for the development of autoimmune DM^36–38^. As DM is a slow progress disease, remaining asymptomatic for a long preclinical period, serological testing is of importance to establish a preventive treatment.

RBA is the reference method for the detection of GADA. Yet, this method is costly, time-consuming and environmentally harmful. For these reasons, non-radiometric methods that are easy to implement for large-scale screening should be developed. To that end, production laboratories should rely on a source of properly folded human GAD65.

There is a very wide range repertoire of expression systems available for the production of foreign recombinant proteins, such as bacterial, yeast, insect and mammalian cells. The choice of the best expression system for a particular protein is related to the yield and the biological or immunochemical activity of the recombinant protein produced. Herein, we report the production of recombinant human GAD65 in insect larvae using the baculovirus expression system. Although GAD65 has been successfully expressed in insect cells cultures, the enzyme was obtained with low yields and purity^16,17,39^. This is the first report on the production of properly folded GAD65 in Lepidoptera larvae. The production system presented herein afforded to obtain high yields and purity. The baculovirus expression system has proved to be a very powerful system for the production of large quantities of biologically active proteins. Besides, this system does not present the drawbacks related to prokaryotic expression systems, such as misfolding and subsequent aggregation to yield inclusion bodies, which turns the protein insoluble and of little value in immunochemical tests based on conformational recognition. It is known that the baculovirus-mediated expression in *Sf*9 insect cells renders proteins with the correct post-translational modifications^40^. On the other hand, insect larvae have scarcely been employed in the production of recombinant proteins.

In the present work, we describe the expression of full length human GAD65 fused to a histidine hexapeptide moiety at the N-terminus, using the baculovirus expression system in two species of larvae. The recombinant baculovirus AcHis_6_-GAD65 was first generated in quantities of roughly10^8^ pfu/mL. *Sf*9 insect cells were employed in the first attempt to express GAD65. As this first approach was successful, insect larvae (*R*. *nu* and *S*. *frugiperda*) were then infected and protein expression analyzed 4 days post-infection. The His_6_ moiety allowed the purification of the recombinant proteins by Ni^2+^-chelating chromatography. The GAD65 expression level in *Sf*9 cells was 3.6 mg/L culture medium, similarly to that reported by Mauch *et al*.^16^, while its expression levels were 1.06 mg per g of larva and 5.7 mg per g of larva in *R*. *nu* and *S*. *frugiperda*, respectively. Thus, the expression level reached in *S*. *frugiperda* was higher than that reached for other recombinant proteins previously produced with the same expression system for diagnostic purposes: DomIIIHFBI (4.5 mg per g of larva)^41^, and InfluenzaA H1N1 neuraminidase (1.2 mg per g of larva)^42^.

The first quality control step for the expressed proteins was the analysis of their enzymatic activity, which directly correlates with the acquisition of the native conformation nearby the active site during the biosynthesis of the enzyme. It is known that GAD65 catalyzes the α-decarboxylation of L-glutamic acid to render GABA. This reaction was evidenced through a colorimetric assay in which the pH of the reaction medium changed due to the consumption of protons. As described in the Results section, both recombinant GAD65 from *R*. *nu* and *S*. *frugiperda* showed enzymatic activity, which was expressed as μmol of glutamic acid consumed to generate GABA per minute and per mg of protein involved in the reaction. TrxGAD, previously expressed by our laboratory in *E*. *coli*, was used as positive control in the assay^35^.

Taking into account the results obtained by SDS-PAGE and WB, together with those of enzymatic activity, it can be assumed that, in this system, genuine human GAD65 was obtained. However, considering that most sera from diabetic patients only target conformational discontinue epitopes in autoantigens, the native conformation of *Sf*9-GAD65, *R*. *nu*-GAD65 and *S*. *frugiperda*-GAD65 was also evaluated with 8 GADA positive sera obtained from type 1 diabetic patients. All 8 positive sera became virtually negative when incubated in the presence of an excess of GAD65 either from *Sf*9 cells, *R*. *nu* or *S*. *frugiperda* (Fig. 2). In addition, dose-response curves generated by RIA with 5 different GADA positive sera showed similarity between *R*. *nu*-GAD65 and *S*. *frugiperda*-GAD65 (Fig. 3), according to the general principles of immunochemical cross-reactivity^43^. These immunochemical studies allowed us to demonstrate that the proteins expressed in both larvae species are able to displace the binding of radiolabeled GAD65 from GADA, thus confirming the integrity of conformational epitopes in all the recombinant antigens produced. As stated above, this is a critical requirement for GADA recognition. It is noteworthy that the approximate median K_a_ values obtained for the 5 sera used in this analysis were in the order of 10^7^ M^−1^ for both, *R*. *nu*-GAD65 and *S*. *frugiperda*-GAD65, which correlates with reported data (range from 10^7^ to 10^11^ M^−1^)^44–46^. No significant differences were found between the K_a_ values for the recombinant GAD65 expressed in both larvae species, suggesting that both enzymes have immunochemical homology.

Full length human GAD65 was successfully expressed as a properly folded protein through a baculovirus expression system in two different hosts, *R*. *nu* larvae and *S*. *frugiperda* larvae. Although the recombinant proteins were obtained with high purity (>95%), and were found to be immunochemically recognized by specific autoantibodies, *S*. *frugiperda*-GAD65 yielded the highest levels of expression (>5 mg GAD65 per g larva). Thus, we decided to evaluate its implementation in the development of low cost immunoassays, such as ELISA for the detection of GADA, either to confirm autoimmune diabetes or for detection in routine screening of individuals at risk of autoimmune DM. For this purpose, we selected 44 type 1 diabetic patients previously subjected to GADA assessment by the reference method RBA, which shows the best sensitivity and specificity parameters in international quality controls (such as the Diabetes Autoantibody Standardization Program –DASP- and Islet Autoantibody Standarization Program –IASP-). The b-ELISA developed is based on the immobilization of the antigen (*S*. *frugiperda*-GAD65) to the solid phase, the incubation with the specific autoantibody (crosslinking molecule) and a fluid phase interaction with the biotinylated antigen (*S*. *frugiperda*-GAD65-biotin) through the available paratope^30,47^. In this case, the b-ELISA for the detection of GADA presented a wide dynamic range (−1.21 to 84.47 SDs), even higher than that described for the reference method (3.04 to 28.21 SDs). The parameters of relative sensitivity (77.3%) and specificity (98.2%) were good enough to be used as a first line screening method for assessing GADA, and are even higher than those obtained for the same type of test in international quality controls (IASP 2015). The thermodynamic principles applied to ELISA and RBA are different; in the latter, the antigen-antibody reaction occurs in the liquid phase, at high dilutions and near the equilibrium state, while in ELISA, the interaction occurs in a solid phase, in which the amount of immunocomplexes is highly dependent on the concentrations of antibodies. Despite these differences, the correlation between both methods was 96.7%, with a kappa index of 0.9302, signifying an excellent agreement. Moreover, the AUC value achieved for b-ELISA (0.8904) was considered highly acceptable (Fig. 4). It is also important to highlight that the amount of recombinant GAD65 recovered from a single larva is sufficient to perform 100 tests of b-ELISA.

Taken together, the results obtained herein support the applicability of *S*. *frugiperda*-GAD65 in the development of b-ELISA for routine determination of GADA. Further studies carried out in our laboratory will assess this recombinant protein in tolerance assays to evaluate prevention strategies of the disease.

In conclusion, this is the first time that human GAD65 is expressed as a recombinant protein in insect larvae. The platform based on *S*. *frugiperda* larvae offers several advantages such as obtaining a biologically active and properly folded product through a simple and unexpensive method. Moreover, this system allows high mass production of GAD65 to be used not only as standard for laboratory research, but also as an antigen for DM diagnosis and treatment.

## Electronic supplementary material

Supplementary Figure 1
