# Supplementary material for: Expression of recombinant glutamic acid decarboxylase in insect larvae and its application in an immunoassay for the diagnosis of autoimmune diabetes mellitus
Source: Sci Rep. 2019 Jan 29;9:824. doi: 10.1038/s41598-018-35744-2 (PMC6351654; doi:10.1038/s41598-018-35744-2)
Supplement: Supplementary file 1 — Supplementary Figure 1 [file 41598_2018_35744_MOESM1_ESM.pdf]

Expression of recombinant glutamic acid decarboxylase in insect larvae and its application in an immunoassay for the diagnosis of autoimmune diabetes mellitus

Aldana Trabucchi, Silvina S. Bombicino, Alexandra M. Targovnik, Juan I. Marfía, Adriana V. Sabljic, Natalia I. Faccinetti, Luciano L. Guerra, Ruben F. Iacono, María V. Miranda, Silvina N. Valdez

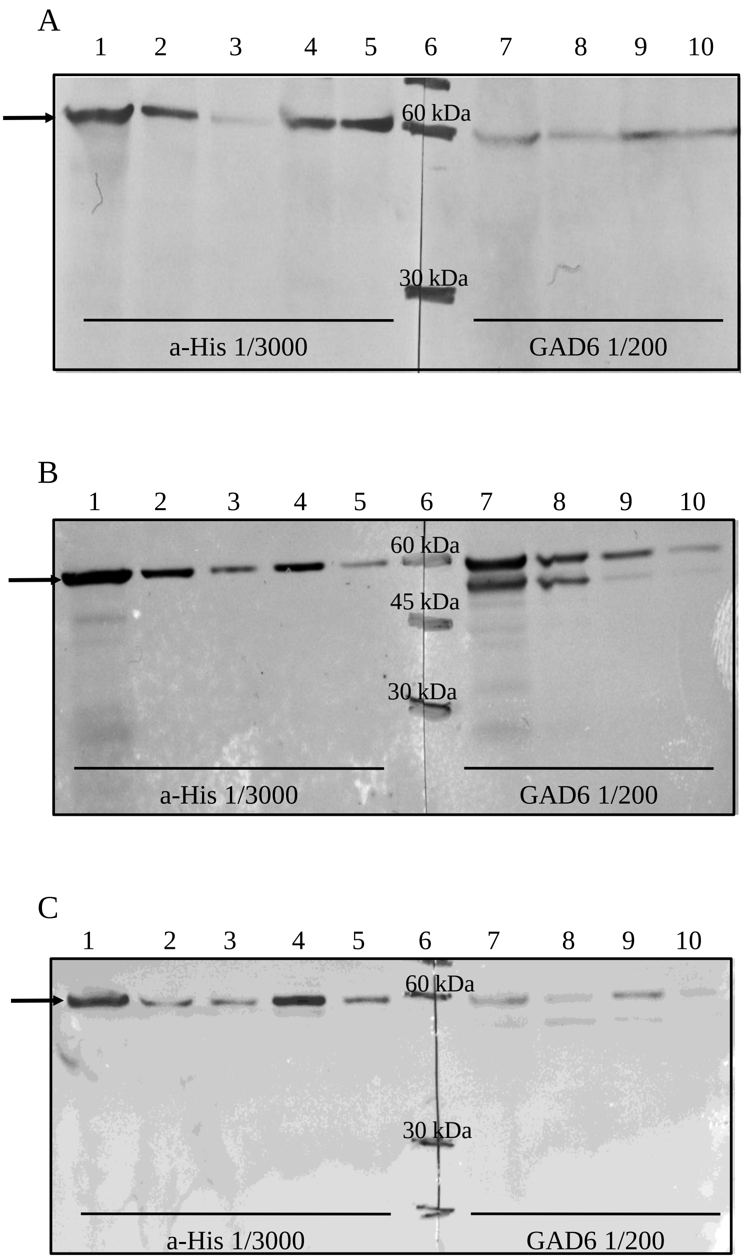

Supplementary Figue 1. WB of GAD65 expression and purification. WB revealed with monoclonal antibodies to His_6 or GAD65 as primary antibodies. Samples: 1. Total soluble fraction, 2. Unbound material, 3. Wash step, 4-5. Consecutive eluates of purified GAD65, 6. MWM, 7. Total soluble fraction, 8. Unbound material, 9-10. Consecutive eluates of purified GAD65. (A) Expression and purification in Sf9-cells, (B) in *R.nu* and (C) in *S.frugiperda*. Arrows indicate the electrophoretic mobility of GAD65.
